# Paucity of HPV-Related Head and Neck Cancers (HNC) in Nigeria

**DOI:** 10.1371/journal.pone.0152828

**Published:** 2016-04-06

**Authors:** Emmanuel A. Oga, Lisa M. Schumaker, Biodun Sulyman Alabi, Darlington Obaseki, Aniefon Umana, Ima-Abasi Bassey, Godwin Ebughe, Olabode Oluwole, Teniola Akeredolu, Sally N. Adebamowo, Patrick Dakum, Kevin Cullen, Clement A. Adebamowo

**Affiliations:** 1 Department of Epidemiology and Public Health, University of Maryland School of Medicine, Baltimore, Maryland, United States of America; 2 Office of Research, Training and Strategic Information, Institute of Human Virology Nigeria, Abuja, FCT, Nigeria; 3 Marlene and Stewart Greenebaum Cancer Centre, University of Maryland School of Medicine, Baltimore, Maryland, United States of America; 4 Department of Ear Nose and Throat, Head & Neck Surgery, University of Ilorin Teaching Hospital, Ilorin, Kwara State, Nigeria; 5 University of Benin Teaching Hospital, Benin, Edo State, Nigeria; 6 University of Calabar Teaching Hospital, Calabar, Cross River State, Nigeria; 7 University of Abuja Teaching Hospital, Abuja, FCT, Nigeria; 8 Department of Nutrition, Harvard T. H. Chan School of Public Health, Boston, Massachusetts, United States of America; 9 National Human Genome Research Institute, National Institutes of Health, Bethesda, Maryland, United States of America; University of Cincinnati College of Medicine, UNITED STATES

## Abstract

**Introduction:**

The burden of HPV-related Head and Neck Cancers (HNC) has been rising in the U.S. and other developed countries but this trend has not been reported in Africa. Objective of study was to evaluate the prevalence of HPV infection in HNC cancer cases seen between 1990 and 2011 at the tertiary health care institutions in Nigeria.

**Methods:**

We retrieved 149 head and neck cancer formalin fixed, paraffin embedded tumor specimens diagnosed between 1990 and 2011 from four teaching hospitals in Nigeria. One hundred and twenty-three blocks (83%) contained appropriate HNC for analysis while DNA extraction was successful in 60% (90/149). PCR amplification was successful in 33% (49/149) and Linear Array genotyping for HPV was successful in 11% (17/149) of these cases. These were in tumors from the larynx (6), cervical lymph nodes (3), nasal cavity (2), parotid (1), palate (1), maxillary sinus (1) and mandible (1). Two cases were non-specific and none were from the oropharynx. Histologically, 41% (7/17) of the successfully genotyped blocks were squamous cell carcinomas (larynx 6, maxillary sinus 1).

**Results and Conclusion:**

We were unable to detect HPV in any of the HNC samples in our study. Our result may suggest that there is a low prevalence of HPV-related HNC among the adult population in Nigeria. Our results provide a benchmark to compare future incidence of HPV -related HNC in this community in future. We had significant analytical challenges from possible poor tissue processing and urge that future studies should prospectively collect samples and ensure high quality sample processing.

## Introduction

Head and Neck Cancers (HNC) are a significant public health concern globally with worldwide Age Standardized Incidence Rate (ASR) of 9.1 per 100,000 population according to GLOBOCAN 2012 data, 10.7 per 100,000 in the United States and 9.0 per 100,000 population on the African continent[1]. While the term HNC could be used to refer to any cancer occurring anywhere from the base of the skull to the clavicles, conventionally it typically refers to cancers of the oral cavity, oropharynx, nasopharynx, hypopharynx, and larynx. Cancers at these locations account for 3% of all cancers in the United States and 4 percent of all cancers globally [1–4].

Risk factors for HNC include consumption of alcohol and smoking and their joint effect can be synergistic [5–7]. Other risk factors are infection by oncogenic viruses including Epstein-Barr Virus (EBV), Human Papilloma Virus (HPV), HIV and Herpes Simplex Virus (HSV); chewing of betel nut, occupational exposure to toxins, radiation; diet, oral hygiene and genetic factors[8].

As much as 25% of all cases of HNC globally are related to high risk HPV (hrHPV). Persistent infection with hrHPV is now accepted to be a major risk factor for the development of HNC, almost exclusively in the oropharynx. According to the International Agency for Research on Cancer (IARC), HPV types 16, 18, 31, 33, 35, 39, 45, 51, 52, 56, 58 and 59 are carcinogenic, and are responsible for all cancers of the cervix and a varying proportion of HNCs[9]. Of all hrHPV types implicated in HNC, HPV 16 alone is responsible for almost 90% of all HPV-related oropharyngeal carcinomas, with HPV 18 a distant second [1, 9–14]. HPV-related HNC is now considered a separate disease entity with different molecular properties, risk factors, clinical manifestation, treatment and prognosis [15–19].

The incidence of HNC is rising throughout the world and most of this rise is attributed to increasing prevalence of hrHPV infection [15, 20]. hrHPV is highly transmissible and most oral infections are cleared within a year[21], although clearance for oral infections depends on several factors like hrHPV type, gender, number of oral sex partners, age, and smoking status[22]. The rise in prevalence of oral hrHPV infections has been primarily linked to changing sexual behaviors particularly the increasingly common practice of oral sex. It has been hypothesized that this is responsible for an increase in incidence of oral hrHPV infection and hrHPV -related HNC. In addition, high number of oral sex partners, multiple vaginal sex partners, younger age at sexual debut, anogenital warts and consumption of marijuana have been implicated in the rising incidence of HNC[23].

Studies in the United States have shown that the burden of hrHPV-related Head and Neck Cancers (HNC) is lower in African Americans compared to white Americans[24–28], and most of the recent increase in incidence has been seen largely in white Americans[29, 30]. Few studies have been done to quantify the burden of hrHPV-related HNC in Africans. In this study, we evaluated the prevalence of hrHPV infection in HNC cases diagnosed at 4 tertiary health care centers in Nigeria between 1990 and 2011.

## Methods

In this multi-center cross-sectional study in Nigeria, HNC tissue samples fixed using 10% Neutral Buffered Formalin (NBF) and embedded in paraffin blocks were used. We retrieved clinical data and Formalin Fixed, Paraffin-Embedded (FFPE) blocks of malignant head and neck tumors diagnosed between 1990 and 2011, from the Pathology Departments of 4 tertiary health institutions in Nigeria: University of Benin Teaching Hospital (UBTH) and University of Calabar Teaching Hospital (UCTH) in Southern Nigeria; including University of Abuja Teaching Hospital (UATH) and University of Ilorin Teaching Hospital (UITH)–both in North-central Nigeria. The tumor blocks were shipped to the United States where pathologists at the University of Maryland Greenebaum Cancer Center reviewed them to confirm the diagnosis. After confirmation of the histological diagnosis, we took sections from the tumor blocks and extracted DNA from these sections using QIAamp DNA FFPE Tissue Kit^®^. We tested for HPV types using Roche Linear Array^®^ HPV Genotyping Test according to manufacturer’s instructions. Ethical approval for study was obtained from the National Health Research Ethics Committee of Nigeria (NHREC). The study used anonymized data from which patient identifiers had been removed prior to analysis.

## Results

Of the 149 tumor blocks retrieved (see Table 1), most, 123 (83%), were eligible for analysis—of the ineligible 26 blocks, 18 blocks had no identifiable tumors, diagnosis was uncertain for 5 blocks, 2 blocks contained uterine tissue and 1 block contained a pleomorphic adenoma of the nasal septum. DNA extraction and purification was successful in 60% (90/149) and PCR amplification was successful in 33% (49/149) and Linear Array genotyping was successful in 11% (17/149) of these cases (See Table 2). Mean age (SD) of patients in samples tested for HPV DNA was 43.3 years (20.27), 10 were males, 6 were females; and mean age (SD) of tissues was 4.0 years (1.54), (See Table 3). At histological review of these 17 tumors, 6 were from the larynx, 3 from cervical lymph nodes, 2 from nasal cavity, 1 parotid, 1 palatine, 1 maxillary sinus 1 from the mandible while 2 were non-specific head and neck cancers (See Table 4). None of the tumors were from the oropharynx. Morphologically, 41% (7/17) of the successfully genotyped blocks were squamous cell carcinomas (larynx 6, maxillary sinus 1). None of the cancers were found to be positive for HPV of any type.

**Table 1:** Characteristics of Samples (N = 149).

**Age of Patient in Years**	
Mean (SD)	43 (20.10)
**Gender, n (%)**	
Male	89(63.60)
Female	51(36.40)
**Age of Tissue Blocks in Years**	
Mean (SD)	6 (3.12)
**Site, n (%)**	
Nasopharynx	26 (18.31)
Larynx	24 (16.90)
Nasal Cavity	21 (14.79)
Cervical Lymph Node	7 (4.93)
Mandible	7 (4.93)
Parotid Gland	6 (4.23)
Sinonasal	5 (3.52)
Thyroid	5 (3.52)
Oral Cavity	6 (4.23)
Palate	4 (2.82)
Oropharynx	3 (2.11)
Parapharynx	3 (2.11)
Lower Lip	2 (1.41)
Maxilla	2 (1.41)
Oesophagus	2 (1.41)
Others	19 (13.38)
**Diagnosis, n (%)**	
Squamous Cell Carcinoma	63 (44.37)
Nasopharyngeal Carcinoma	25 (17.61)
Undifferentiated Carcinoma	6 (4.23)
Ameloblastoma	5 (3.52)
Metastatic Carcinoma	5 (3.52)
Mucoepidermoid Carcinoma	5 (3.52)
Adenoid Cystic Carcinoma	4 (2.82)
Kaposi Sarcoma	4 (2.82)
Anaplastic Carcinoma	3 (2.11)
Burkitt's Lymphoma	3 (2.11)
Papillary Carcinoma	3 (2.11)
Follicular Carcinoma	2 (1.41)
Rhabdomyosarcoma	2 (1.41)
Spindle Cell Carcinoma	2 (1.41)
Others	10 (7.04)

**Table 2:** Outcomes of Molecular Analysis.

Study Site	Calabar	Illorin	Benin	Abuja	Total
Blocks provided	46	41	28	34	149
Suitable for DNA core	42	32	20	29	123
DNA concentration>1ng/μl	41	25	18	6	90
Genotyping success (attempted)	11 (32)	4 (7)	2 (6)	0 (0)	17 (45)
HPV positive	0	0	0	0	0

**Table 3:** General Characteristics of Samples Tested for HBV DNA (n = 17).

Study Site	Calabar	Illorin	Benin	Total
Gender: M, F	7, 3	2, 2	1, 1	10, 6
Age (years): Mean (SD)	39.2 (19.2)	43.8 (24.0)	62.5 (14.9)	43.3 (20.27)
Age of Tissue Blocks (years): Mean (SD)	5.0 (2.0)	4.0 (1.0)	5.0 (1.0)	4.0 (1.54)

**Table 4:** Distribution by Anatomical Site and Pathological Diagnosis of Final Sample (n = 17).

Anatomical Site	n (%)	Pathological Diagnosis	n (%)
Larynx	6(35.29)	Anaplastic Carcinoma	1 (5.88)
Cervical Lymph Nodes	3(17.65)	Squamous Cell Carcinoma	7 (41.17)
Nasal Cavity	2(11.77)	Metastatic Carcinoma	2 (11.77)
Palate	1(5.88)	Non-Hodgkin Lymphoma	2 (11.77)
Maxillary sinus	1(5.88)	Adenoid Cystic Carcinoma	2 (11.77)
Others	4(23.53)	Others	3 (17.65)

## Discussion

In this retrospective study of HNC tumor blocks from 4 referral hospitals in Nigeria, we found that out of the 149 FFPE blocks available for studies, we did not identify any Oropharyngeal cancers; HPV genotyping was successful in 17 (11%) cases and none of these were positive for HPV DNA. We also did not find any tumor that specifically originated from the oropharynx in this sample. Despite studies from other parts of the world that indicate that the Oropharyngeal cancers account for a huge proportion of all HPV-related HNC[2, 4], our result suggest that hrHPV-related HNC has not yet emerged as a significant health burden in the Nigerian population. Another study from north-central Nigeria reveals that most of the HNC treated at a tertiary health center there were nasopharyngeal carcinomas while Oropharyngeal cancers were not as common as expected, accounting for only about 7%[31]. Other reports show a similar paucity of oropharyngeal carcinomas in Nigeria [32–34].

The low prevalence of hrHPV-related HNC among Nigerians may be due to several factors. hrHPV -related HNC has been associated with practice of oral sex and the prevalence of this sexual behavior has been rising among youths and young adults with a recent study showing that oral sex practice, though widely common, is more prevalent in younger age groups[35–37]. In the U.S., studies suggest that whites are much more likely to report having had more than 5 oral sex partners than blacks and Hispanics of both sexes; whites are also at least 3 times less likely than blacks and Hispanics to report never having had oral sex. Consequently, oral hrHPV infection is commoner among whites compared to African Americans and this may explain the higher prevalence hrHPV-related HNC in white Americans [37–39].

Furthermore, the practice of oral sex appears to be less common among Africans than in Americans of any race. Studies on adolescents for example, shows that prevalence of having ever had oral sex for African adolescents ranges from 5.4%, to 13.3% compared with > 75% in American adolescents, population-wide studies of oral sex behavior in Africans shows a prevalence of less than 12% compared with at least 70% in Americans of all ethnicities[37, 38, 40–43]. The relatively lower prevalence of oral sex practice among Africans may be one reason why hrHPV -related HNC occurs less frequently in this population.

The difference in incidence of hrHPV -related HNC may also be related to genetic differences between Africans and White Americans. Ndiaye *et al*[44] showed that the burden of HPV -related HNC is lower in African-Americans than in white Americans, even after controlling for sexual behavior and age although this may be due to residual confounding[38]. Consequently, some have suggested that there are innate differences in host-virus interactions between whites and blacks which may account for differential impact of hrHPV infection on the etiology of HNC[45]. There is also suggestion that the microbiome may be a factor in persistent HPV infection of the uterine cervix, it is possible that a similar mechanism is at play with regard to the oral microbiome and oral HPV infection[46].

There was a high prevalence of inadequate tumor samples for molecular biology analysis and HPV genotyping in this study, which may reflect poor infrastructure for tumor pathology in Nigeria. A study of HNC FFPE tumor blocks in South African blacks with oropharyngeal cancer detected HPV DNA in 94.1% of cases [47]. This result differs from previous Africans studies, including 3 prior studies in South Africa that show prevalence of HPV at 1.4%, 1.5% and 11.5% [48–50]. Furthermore, a study HNC FFPE tumors from Senegal, found HPV DNA in only 3.4% of cases[44]. There is no reason to suspect that hrHPV -related tumors would be more difficult to analyze than HNC that are not associated with hrHPV. Fresh frozen biopsies would yield better HPV detection rates than FFPE but the logistics of obtaining these types of tumors in Africa is quite challenging[9]. Other potential challenges of HPV DNA detection from FFPE include barrier effect of paraffin, DNA cross-linkages due to formaldehyde and tissue fragmentation[51].

There has been significant variation in prevalence of hrHPV-related HNC between studies that utilized different HPV detection techniques. Higher prevalence of HPV has been reported in HNC when PCR methods were used instead of *in situ* hybridization, making it even more complicated to pool results from several studies[19]. Different assay techniques are available and all having different levels of sensitivities when used for HPV identification. Results can also vary depending on the anatomic origin of tumor [52, 53].

Our study suffers from several limitations. The quality of fixation was suboptimal in a significant proportion of the tumor blocks and DNA detection (tumor availability, DNA recovery, purification, amplification and genotyping) was not successful for many samples. Attempts to repair formaldehyde-induced DNA damage did not yield meaningful success. Future studies of HNC and hrHPV -related HNC in Nigeria should be done prospectively and processed in good quality laboratories with standard QA/QC procedures in order to ensure that the resulting tumor blocks are amenable to molecular biology studies. Our sample size was small and this may be a reflection of the low incidence of HNC in our population. Many patients may also present late or do not present at all to hospital and die before a definitive diagnosis of HNC is made[54].

In conclusion, we did not find any case of hrHPV-related HNC in our sample. While this is probably a result of low prevalence of HPV-related HNC, our results should be interpreted cautiously because of the small effective sample size and low DNA yield.

## Supporting Information

S1 Table Dataset. (XLSX)
